# A Systematic Review of Canine Cystectomy: Indications, Techniques, and Outcomes

**DOI:** 10.3390/ani13182896

**Published:** 2023-09-13

**Authors:** Isabella Hildebrandt, William T. N. Culp, Maureen A. Griffin

**Affiliations:** 1 Department of Clinical Sciences and Advanced Medicine, School of Veterinary Medicine, University of Pennsylvania, 3800 Spruce St., Philadelphia, PA 19104, USA; 2 Department of Surgical and Radiological Sciences, School of Veterinary Medicine, University of California-Davis, One Garrod Avenue, Davis, CA 95616, USA

**Keywords:** canine, cystectomy, urothelial carcinoma, transitional cell carcinoma, lower urinary tract neoplasia

## Abstract

**Simple Summary:**

This review provides a summary of the literature encompassing partial and complete bladder removal surgeries in dogs and subsequent conclusions that can be drawn. Removal of bladder tumors as a component of treatment for cancer of the lower urinary system in dogs may enhance survival time and result in acceptable quality of life, though risk for complications is substantial, particularly following removal of the entire bladder. However, for dogs with urothelial carcinoma, surgical removal of the tumor is generally not considered curative and disease progression is common. Appropriate case selection and thorough discussion with owners regarding potential risks and benefits of surgery are imperative for successful outcomes.

**Abstract:**

This review provides a summary of the literature encompassing partial and total cystectomy procedures in dogs and subsequent conclusions that can be drawn. Surgical excision as a component of treatment for lower urinary tract neoplasia in dogs may enhance survival time and result in acceptable quality of life, though risk for surgical complications is substantial, particularly following total cystectomy procedures. However, for dogs with urothelial carcinoma, cystectomy is generally not considered curative and disease progression is common. Appropriate case selection and thorough preoperative discussion with owners regarding potential risks and benefits of cystectomy are imperative for successful outcomes.

## 1. Introduction

The urinary bladder is the most common site for urinary tract neoplasia in dogs [1]. Urinary bladder neoplasia accounts for approximately 2% of all canine malignancies, with urothelial (transitional cell) carcinoma being the most prevalent [2,3,4,5,6,7,8]. Urothelial carcinoma (UC) most frequently occurs at the trigone with common involvement of the urethra (>50%) and prostate of male dogs (29%) [1,3,4,9]. Local extension from urethral or prostatic tumors into the urinary bladder is common but metastasis to the urinary bladder from distant locations is rare [4,10,11]. Other reported canine urinary bladder neoplasms include lymphoma, embryonal rhabdomyosarcoma, adenocarcinoma, squamous cell carcinoma, hemangioma, hemangiosarcoma, fibroma, fibrosarcoma, osteosarcoma, leiomyoma, and leiomyosarcoma [2,8,11,12,13,14,15].

Urothelial carcinoma tends to be an aggressive, invasive tumor with a high rate of metastasis. As with many tumor types, the World Health Organization (WHO) TNM stage at the time of diagnosis has been strongly associated with prognosis [7,8]. According to this staging scheme, 78% of dogs with UC have T2 stage tumors that invade the bladder wall and 20% have T3 stage tumors that invade adjacent structures [8,16,17]. It has been reported that at the time of diagnosis, 16% of dogs have nodal metastasis, 14% have distant metastasis, and 10% have both nodal and distant metastasis [2,4,8]. A study evaluating stage upon necropsy reported an overall metastatic incidence of 67%, with nodal metastasis in 42% and distant metastasis in 58% of dogs with UC [7,8]. The lungs are reportedly the most common site of distant metastasis (50%), and bone metastasis has also been documented in 9% of dogs [7,18]. Despite the high incidence of metastasis, progression of local disease with urinary tract obstruction is the cause of death for many dogs if the primary tumor is not adequately controlled [4]. In dogs with UC that have a known cause of death, it has been associated with the primary tumor in 61% and with metastasis in 14% [4]. However, if local disease control is achieved, metastatic disease becomes the most common cause of death [4]. These results stress the importance for both local and systemic treatment in dogs with UC.

With currently available therapies, canine UC is not generally considered a curable disease but can be controlled in at least 75–80% with a good quality of life for several months to greater than 1 year, with a small proportion of dogs living longer than 3 years [2,8,9]. Medical therapy, surgery, radiation therapy, and local intravesical therapy are reported treatments for UC in dogs, and palliative surgical and interventional treatments can be performed to alleviate obstructions associated with UC. Systemic medical management is the mainstay treatment for canine UC [9,11]. A variety of protocols have been described involving multiple combinations of cyclooxygenase (COX) inhibitors and chemotherapy agents. The current medical therapy recommendation is treatment with sequential administration of multiple drugs over the course of disease, with alteration of protocols at the time of disease progression and/or toxicity, as UC is generally considered relatively chemotherapy resistant [2,7,8].

Canine UC shares many similarities with high-grade invasive UC in humans, though this neoplasm has a more balanced distribution of bladder locations in humans as compared to dogs [7,9]. Dogs with UC serve as an important spontaneous model for invasive UC in humans due to the similar microscopic and molecular features, biologic behavior, and response to treatment [9]. Translational studies allow for development of innovative diagnostics and treatments that thereby serve both human and veterinary patients with UC [7,9]. For humans with invasive UC confined to the bladder, radical cystectomy and neoadjuvant chemotherapy are the mainstay local and systemic treatments, respectively [9,19,20]. However, in dogs with UC, radical cystectomy has not gained widespread acceptance as a mainstay local treatment modality due to the common trigonal location, frequent extension of disease beyond the bladder (both local and systemic), potential for incomplete excision even with radical cystectomy, possible “field effect” with diffuse malignant transformation of the urothelium and subsequent local progression, complications associated with the procedure, and expense involved [2,4,8,9,11,21,22]. Therefore, in dogs with UC, surgery has predominantly shifted from curative intent to palliative intent with the goals of improving quality of life and maintaining patency of the urinary tract. However, multiple recent reports have demonstrated novel techniques for cystectomy procedures in dogs with relatively good outcomes and owner satisfaction. This review aims to summarize the cystectomy literature in dogs and draw conclusions regarding indications, techniques, morbidity, and outcomes.

## 2. Partial Cystectomy

### 2.1. Indications and Case Selection

Partial cystectomies were initially described in 1996 by Stone et al. for 11 dogs with bladder neoplasia [23]. There have subsequently been multiple reports of dogs receiving partial cystectomies for treatment of UC, leiomyoma, leiomyosarcoma, hemangiosarcoma, and non-neoplastic conditions [14,24,25,26,27,28,29,30,31].

Most commonly, cystectomy procedures are considered for local treatment of dogs with solitary, localized disease without evidence of metastasis on staging diagnostics. Traditionally, partial cystectomies have been predominantly performed in dogs with solitary tumors that are amenable to wide excision and primary closure of the bladder [32]. These discrete lesions are generally located distant from the trigone of the bladder at the apex, craniodorsal wall, or cranioventral wall [14,24,25,28,29,31]. However, in several studies, partial cystectomies have been performed for tumors located in the trigone and neck of the bladder involving the proximal urethra or the ureterovesicular junctions [15,23,24,25,30,33]. For these cases in which the communications between bladder and urethra/ureters were preserved, surgical margins were generally not wide relative to the tumor due to proximity of the trigone and associated structures. However, if wide excision is pursued for tumors in these locations, or if resection of all gross disease inherently involves the ureterovesicular junction(s) or urethra, additional procedures such as neoureterocystostomy and/or bladder-urethral anastomosis become necessary [15,23,24,25,33]. It is important to also consider the potential for compromise of the neurovascular supply (particularly the caudal vesical artery and pelvic plexus, comprising somatic, parasympathetic, and sympathetic innervation) to the bladder, which enters dorsally at the bladder neck near the trigone. If this pedicle is not able to be preserved and the bladder is inadvertently devitalized during planned partial cystectomy for a tumor near the trigone, radical cystectomy may be required; if the vascular supply is preserved but the neurologic supply is damaged, permanent incontinence may result. Dissection and preservation of the neurovascular supply for partial cystectomy of trigonal tumors requires that the tumor is not grossly invading the serosa/muscularis of this dorsal trigonal region where the neurovascular pedicle enters the proximal urethra and bladder [15].

As described in detail below, there are reports of use of a bipolar sealing device (BSD) for partial cystectomy in dogs [34,35]. Although these devices are not designed for sealing hollow viscous organs, they have been effectively utilized for cystectomies in humans [36,37]. Use of a BSD is limited to cases with tumors in a non-trigonal bladder location. If concurrent cystoliths are present that cannot be evacuated via hydropulsion, a standard partial cystectomy is indicated as use of a BSD does not allow for bladder opening and stone removal [34].

Aside from location of the bladder tumor, an additional important consideration involves tumor size and amount of residual bladder that will remain following resection. Large partial cystectomies can result in pollakiuria and potentially urinary incontinence, and owners’ willingness to manage these sequelae should be considered and discussed preoperatively.

An alternative consideration for partial cystectomy involves partial thickness excisions of bladder tumors. This is mainly indicated for benign mucosal lesions, as in dogs with multiple polyps throughout the bladder resulting in clinical signs [38]. Though partial thickness resection can be considered for cytoreductive palliation of UC causing urinary obstruction, this is often not feasible without leaving residual gross disease owing to the invasive nature of these tumors.

### 2.2. Surgical Techniques: Partial Cystectomy

#### 2.2.1. Surgical Techniques without Reconstruction/Augmentation

All surgical cystectomy techniques require a ventral midline approach to the abdomen (caudal celiotomy) and adequate isolation of the bladder from the abdominal cavity. The serosal layer of the bladder should be examined for any gross abnormalities and blood supply. Tumor identification can be performed via external palpation of the bladder and/or concurrent cystoscopy [23,34,39]. Intraoperative visual inspection and palpation of the entire urinary bladder (both serosal and mucosal surfaces, provided the bladder is opened), as well as palpation of the proximal urethra, should be performed to evaluate for gross extent of neoplastic disease.

For a standard cystectomy, fine-gauge monofilament stay sutures are placed near the planned cystectomy incision to facilitate bladder handling and manipulation. An initial bladder incision is made with a blade in a location along the planned partial cystectomy site, ideally based on margins of 1–3 cm (depending on the location and size of the tumor as well as the size of the bladder) for wide excision from the gross disease [38]. The incision is then extended with scissors to allow for evaluation of the bladder mucosa and completion of the cystectomy. If the planned surgical margin encroaches on the ureterovesicular junction(s), decision making requires either sacrificing margins to preserve the junction(s) or excising the ureteric orifice with subsequent reimplantation of the ureter(s) [15,23,38]. If the ureterovesicular junction is excised, a margin of at least 2 cm of ureter is generally excised concurrently in an effort to achieve histologically tumor-free margins [15,38]. Following partial cystectomy, bladder wall closure is routine via a one- or two-layer appositional closure with a simple interrupted or simple continuous pattern using fine-gauge, absorbable, monofilament suture material. When closure is adjacent to a ureteral orifice, it is important to avoid entrapment of the ureter with suture. Leak testing can be performed via a needle or urethral catheter following bladder closure. Although some reports document placement of an indwelling urinary catheter at the time of surgery to keep the bladder decompressed during early healing, the need for this is controversial and indwelling catheter placement is often not indicated/required [14,23,31]. Following closure of the urinary bladder, lavage should be performed to remove any blood clots and potential tumor cells, and gloves and instruments should be changed prior to routine abdominal closure in an effort to reduce the risk of seeding tumor cells [38]. Postoperatively, dogs are generally administered intravenous fluids at a maintenance rate to flush out blood clots and debris from the bladder [38]. If a urinary catheter is not placed, frequent walks are indicated given the potential for pollakiuria, especially with large resections [38].

One study described a partial cystectomy technique to treat dogs with lower urinary tract obstruction secondary to trigonal tumors with preservation of the bladder neovascular supply [15]. In two dogs with trigonal tumors and urinary obstruction, the dorsal serosa containing the neurovascular pedicles to the bladder and urethra (grossly disease-free) was dissected and preserved, with ligation of the deeper vasculature, and the bladder neck and proximal urethra were subsequently resected en bloc [15]. In these dogs, resection of the ureterovesicular junctions also occurred and neoureterocystostomies were required [15]. The proximal urethra was anastomosed to the remaining bladder (body) over a catheter via a simple interrupted pattern with 4-0 polydioxanone [15]. The largest study to date on dogs that underwent partial cystectomy for treatment of UC included 5/37 dogs that had partial thickness excisions in the region of the trigone, and 2/37 dogs that required neoureterocystostomies due to trigonal involvement [25]. An additional case report of partial cystectomy involving the trigone of the bladder described a full thickness 40–50% partial cystectomy with resection of the bilateral ureterovesicular junctions and subsequent bilateral neoureterocystostomies [33].

For partial thickness cystectomy procedures, a cystotomy is performed away from the site(s) of planned partial thickness resection(s), and the submucosa is dissected with sharp and blunt dissection or use of a CO_2_ laser to resect the lesion(s) with appropriate margins [38]. The submucosal defect is then closed primarily in a single layer with fine-gauge, absorbable, monofilament suture [38].

The use of a BSD to seal bladder tissue for closure of partial cystectomies has also been described in both cadavers and live dogs with non-trigonal bladder tumors [34,35]. In the live dog study, the bladder was first expressed to decrease intraluminal pressure prior to partial cystectomy with the BSD [34]. The bladder was not opened, and the BSD (LigaSure^TM^; Medtronic, Minneapolis, MN, USA), set at power level 3, was applied in a curvilinear fashion based on the shape and margins of the planned excision [34]. LigaSure handpieces consisted of small jaw (4), Precise (2), and Atlas (1); when using the Precise handpiece, two seal cycles and use of scissors to cut between the seals were required, whereas only a single seal cycle was required for the other handpieces that allowed for cutting with the LigaSure cutting blade [34]. Following BSD sealing in both cadaveric and live dog tissues, gap formation along the BSD seal was noted in a portion of dogs [34,35]. Because of this gap formation and the ex vivo canine cadaveric study finding that augmenting the BSD seal with a suture oversew resulted in higher intraluminal pressure at catastrophic leakage compared with the BSD seal alone, the BSD seal in live dogs was oversewn with 3-0 or 4-0 absorbable monofilament suture (polydioxanone or glycomer 631) in a one-layer simple continuous pattern [34,35]. Importantly, due to the potential for leakage and uroabdomen if suture is placed in tissues within the BSD peripheral thermal effect zone, this oversew line should be placed over the seal in grossly normal bladder tissue [34]. Intraoperative leak tests and placement of indwelling urinary catheters were not performed in these dogs [34].

#### 2.2.2. Surgical Techniques with Reconstruction/Augmentation

Various grafts have been experimentally used to reconstruct bladder defects in dogs, with the goal of bladder tissue regeneration and replacement over time. Most of these studies have evaluated augmentation of the bladder wall following varying extents of partial cystectomy with preservation of the trigone, with substitute tissues used to replace the portion of bladder wall excised. Biodegradable bovine pericardial tissue grafts resulted in bladder enlargement while the graft was progressively resorbed over time and the bladder wall was regenerated to adequate capacity and function [40]. Human placental membranes have also been used for patch augmentation of experimental canine partial cystectomies [41,42]. Although 1 of 14 dogs developed uroabdomen postoperatively, other dogs had evidence of appropriate urothelial regeneration [41,42]. Homologous bladder acellular matrix graft has also been applied following partial cystectomy in experimental dogs, resulting in improved mean bladder capacity and bladder regeneration [43]. A fresh autologous tunica vaginalis graft has also been used to replace the excised portion of bladder wall after partial cystectomies in experimental male intact dogs with subsequent bladder regeneration, though flap shrinkage of 33–56% occurred in all dogs and calcification and bone metaplasia were also observed [44]. Porcine small intestinal submucosa (SIS) has also been used as a bladder augmentation material in experimental dogs undergoing up to 45% partial cystectomy, with resulting normal bladder capacity and function as well as evidence of bladder wall regeneration over time postoperatively [45]. However, when SIS was used as an augmentation material in a canine model of subtotal (90%) cystectomy, limited bladder regeneration occurred, moderate to heavy adhesion, graft shrinkage, and bone and calcification were observed at the graft site, 3/12 dogs developed bladder obstruction by stones (suspected to be secondary to incompletely absorbed SIS), and 1 of 12 dogs developed bladder perforation due to incompletely absorbed SIS [46]. Hypotheses for the lack of regeneration included an inability to develop rapid neovascularization to the graft, inflammation and scarring following subtotal cystectomy, and lack of structural support from the remaining small bladder template [46].

Additional described techniques for urinary bladder augmentation following partial cystectomy, without the end goal of graft replacement and bladder tissue regeneration, include use of colonic seromuscular augmentation and a rectus abdominis muscle flap. One case report exists of a dog that underwent colonic seromuscular augmentation cystoplasty following subtotal cystectomy for treatment of bladder necrosis secondary to torsion at the level of the trigone following ovariohysterectomy [47]. A subtotal cystectomy was performed with preservation of approximately 5 mm of tissue cranial to the ureterovesicular junctions, and the remaining bladder stump was apposed to the antimesenteric serosal surface of the adjacent colon via simple interrupted sutures of 4-0 polydioxanone [47]. Nearly 3 months postoperatively, the dog developed a ureteral stricture and required neoureterocystostomy, and at 13 months postoperatively, the dog developed pyelonephritis [47]. However, the dog’s urinary bladder returned to nearly normal size within 3 months of surgery, and pollakiuria improved to relatively normal bladder function at 4 months postoperatively [47]. Another case report documented use of a rectus abdominis muscle flap [48]. Following debridement of necrosis in the region of the proximal ventral urethra and portion of the bladder trigone (secondary to pressure necrosis associated with a large urethrolith), an axial pattern flap based on the caudal superficial epigastric vessels of the caudal rectus abdominis muscle was raised and sutured to the bladder defect (such that the dorsal aspect of the flap was oriented toward the bladder lumen) with 4-0 polydioxanone in a simple continuous pattern [48]. Cystoscopy was performed 2.5 years after surgery and revealed the flap to be covered with normal-appearing mucosa with no distinguishable junction between muscle and bladder; the dog was clinically doing well with no signs of urinary incontinence [48].

It is important to note that each of these studies included relatively small case numbers. In addition, the regenerative grafting techniques have only been performed in healthy, experimental dogs undergoing partial cystectomy. Also, the native tissue augmentation techniques have only been reported as case reports for dogs undergoing partial cystectomy due to bladder necrosis without evidence of neoplasia. Additional studies and clinical application need to be investigated for use of these techniques in dogs with spontaneous urinary bladder neoplasia and in dogs undergoing both partial and total cystectomy. In general, these techniques are not routinely indicated due to the relatively good functional outcomes with partial cystectomies without bladder wall augmentation/replacement techniques in clinical dogs with bladder neoplasia, as further described below.

### 2.3. Complications and Outcomes

Intraoperative complications are scarcely reported in cases of partial cystectomy. The only noted intraoperative complication in a study on 37 dogs that underwent partial cystectomy for UC was hemorrhage requiring a blood transfusion [25]. The majority of dogs survive to discharge, with deaths in the postoperative period predominantly unrelated to surgery or anesthetic complications [14,23,24,25]. Potential complications associated with partial cystectomy include ureteral/urethral obstruction secondary to blood clots, stricture and/or tumor recurrence, uroabdomen, bladder necrosis, incisional complications, disease progression/recurrence, and neoplastic seeding of the abdomen/abdominal wall [23,25,30,33,34,38,49]. Pollakiuria, stranguria, and hematuria are common transient postoperative findings [23,25]. Bladder necrosis and leakage with uroabdomen have been reported between 2 and 10 days postoperatively [23,30,34,49]. The majority of these cases (3/5) were taken back to surgery for revision cystectomy without additional complications [23,34]. This included a dog that underwent BSD-sealed partial cystectomy with the oversewn suture line performed within the BSD thermal zone; revision cystorrhaphy was performed 3 days postoperatively in this dog without subsequent complication [34]. One study reported diffuse bladder necrosis identified 10 days postoperatively; this was suspected to be due to damage or thrombosis of the caudal vesical artery entering the dorsal bladder surface near the trigone [30].

Approximately 10–11% of dogs have been reported to develop disease due to tumor seeding within the abdominal cavity and/or incision site following partial cystectomy/cystotomy for UC at a median of 121–150 days following surgery [25,50]. However, a recent study including 31 dogs that underwent partial cystectomy for UC documented only 1 dog (2.7%) that developed tumor seeding of the abdominal wall, though postoperative staging was not standardized in this retrospective study [24]. As UC tumor seeding is associated with a poor prognosis, it is important to take efforts to minimize the risk of seeding at the time of surgery as well as during preoperative diagnostics [50]. A potential theoretical advantage of partial cystectomy with BSD involves reduced exposure of the intraluminal bladder contents (containing neoplastic cells) to the abdomen, thereby reducing the risk of neoplastic seeding of the abdomen and/or body wall. However, the requirement for an oversewn suture line placed in the non-sealed adjacent bladder tissue has the potential for seeding tumor cells as well. The effect of BSD partial cystectomy on risk of tumor seeding is not yet known in dogs, though none of seven dogs were reported to have body wall tumor seeding at a median follow-up time of 275 days post-BSD partial cystectomy [34].

Functional outcomes following partial cystectomy in dogs are usually reported as good to excellent [38]. Although partial cystectomy initially reduces urine storage capacity and results in pollakiuria in the majority of dogs, pollakiuria is generally transient and urinary capacity approaches normal within several weeks postoperatively in most dogs [23]. Following cystectomy with resection of 35–40% of the bladder, dogs generally regain baseline bladder storage capacity within 10 months [45]. Even with large bladder resections, most dogs regain acceptable function and can hold urine overnight by 3 months postoperatively [51]. For 11 dogs receiving partial cystectomy with resection of 40–70% of the bladder, no urinary incontinence was noted but 2/11 (18%) dogs experienced pollakiuria [23]. When 90% of the bladder was excised with an intact trigone remaining in 22 dogs, the mean bladder capacity was decreased by 72% from baseline at 9 months postoperatively, with all dogs having pollakiuria [46]. In the study on two dogs that underwent en bloc removal of the bladder neck and proximal urethra with preservation of the neurovascular pedicles, both dogs had appropriate urinary continence and regained normal urinary function after transient postoperative pollakiuria [15].

Overall outcomes vary depending on tumor type, disease stage, and additional treatments performed. One retrospective study on dogs with UC compared 31 dogs treated with partial cystectomy and adjuvant medical therapy (chemotherapy and COX inhibitors) to 16 dogs treated solely with medical management [24]. Median overall survival time for dogs that received surgery and adjuvant medical management was greater (498 days) than for dogs that received medical management alone (335 days) [24]. However, this study found no difference in median progression-free survival time between groups [24]. For 37 dogs that underwent partial cystectomy for UC and various non-surgical treatments, the median survival time was 348 days and the median progression-free interval was 235 days [25]. Prognostic factors associated with increased survival time included age, tumor location, full thickness excision, and frequency of piroxicam administration [25]. Tumor-free histologic margin was not found to be a prognostic factor in either of these retrospective studies [24,25]. When dogs received partial cystectomy and daily piroxicam (+/− chemotherapy), the median survival time was 772 days [25]. The authors concluded that dogs with non-trigonal UC treated with full thickness partial cystectomy and daily piroxicam appear to have improved outcomes relative to historic data on dogs treated without surgical excision as a component of treatment [25]. Rare reports of dogs undergoing partial cystectomy for non-UC tumors also exist. Two dogs with rhabdomyosarcoma were euthanized at 5 and 7 months postoperatively due to local recurrence and pulmonary metastasis, respectively [15,23]. Single cases of dogs with primary bladder hemangiosarcoma and leiomyosarcoma treated with partial cystectomy as a sole therapy have been reported, and the dogs were alive at 9 and 29 months postoperatively, respectively [14,26].

### 2.4. Discussion

Overall, partial cystectomy and primary closure of the bladder is associated with relatively good urinary function, a low complication rate, and potential for improved outcome in dogs with UC. One consideration from the present literature is that the importance of histologically tumor-free margins is not known for partial cystectomy in dogs with UC, as the two largest retrospective studies to date on dogs with UC that underwent partial cystectomy demonstrated no difference in outcomes relative to histologic margin status [24,25]. Recurrence of UC within the lower urinary tract is common following partial cystectomy, even when histologically complete margins are achieved, supporting a potential field cancerization effect or localized seeding of tumor cells within the bladder [2,23,25]. It is therefore important to weigh the risks of morbidity and complications following aggressive partial cystectomy resections with the unknown benefit of achieving histologically tumor-free margins in dogs with UC. Ultimately, partial cystectomy may have a role in improving outcomes for dogs with UC and surgery is predominantly indicated in a setting in which all gross disease can be excised. Based on the evidence provided, preservation of important structures (such as the neurovascular supply to the bladder and bladder communications with the ureters and urethra) should generally be prioritized over performing wide excisions in an effort to obtain histologically tumor-free margins given the lack of demonstrated benefit of histologically tumor-free margins and risk for complications with more aggressive resections.

## 3. Total Cystectomy

### 3.1. Indications and Case Selection

Because canine UC typically occurs in the trigone, partial cystectomy with trigonal excision (+/− neoureterocystostomy and/or urethra-bladder anastomosis) is only possible if preservation of the neurovascular supply to the bladder is feasible [15,38]. Because this is not often possible with locally advanced disease, total cystectomy is required if surgical excision is to be performed for these cases. Total cystectomy is also required for surgical excision of diffuse urinary bladder disease. Total cystectomy requires concurrent urinary diversion and/or bladder replacement, and multiple techniques have been described. Because adverse events are largely associated with the mechanism of urinary rerouting, complications and outcomes have been described in each section relative to surgical reconstruction/urinary diversion technique. Factors that are important to consider relative to case selection include tumor type, disease stage, overall prognosis, risk of complications associated with surgery, alternative non-surgical and palliative treatment options (and the risks and outcomes associated with each of those), and owners’ goals and willingness to manage surgical complications and permanent urinary incontinence.

### 3.2. Surgical Technique, Complications, and Outcomes: Total Cystectomy

For all dogs undergoing total cystectomy, standard ventral midline celiotomy is performed, and the bladder and associated neoplasm must be dissected and excised [38,52,53]. The ureters are generally identified first and transected at an adequate margin from the gross neoplasm, while maintaining adequate length relative to the planned urinary diversion technique. Stay sutures can be placed on the distal ureters, and they can also be temporarily catheterized with small feeding tubes or urinary catheters to facilitate manipulation and urinary diversion procedures. The round ligament of the bladder is dissected to allow mobilization of the bladder. The caudal vesical arteries are ligated and transected near their entrance into the dorsal bladder neck. The urethra is isolated from surrounding tissues, transected, and ligated at a location determined by tumor extension and urinary diversion technique. Prostatectomy can be performed concurrently if the prostatic urethra is involved [54]. The entire bladder, along with proximal urethra and distal ureters, is subsequently removed en bloc. The abdomen should be lavaged to remove blood clots and tumor cells, and gloves and instruments should be changed in an effort to reduce the risk of neoplastic seeding of the abdomen and body wall prior to urinary diversion or bladder reconstruction and subsequent abdominal closure.

Following total cystectomy, permanent postoperative urinary incontinence is generally expected [38]. Potential complications associated with total cystectomy include uroabdomen, surgical site complications (infection, dehiscence, seroma/swelling), and disease recurrence/progression.

### 3.3. Surgical Techniques, Complications, and Outcomes: Bladder Replacement and/or Urinary Diversion Methods

#### 3.3.1. Enterocystoplasty

Different segments of the gastrointestinal (GI) tract have been investigated for bladder substitution following total cystectomy in dogs due to the robust blood supply and ability to distend these organs. Following dissection and isolation (with preservation of blood supply) of the intended GI component to be used as a substitute, the GI segment is anastomosed to the urethra and ureters, and the remaining GI tract is closed and rerouted. However, with these techniques, the segments of GI tract retain characteristics and functions of the native bowel. As such, substitution of the bladder with GI elements results in bacterial colonization, metabolic disturbances due to urine absorption, and native tissue ulceration [55,56,57].

Both the stomach and small intestine have been utilized for neobladder formation in dogs. In an initial study, when the pylorus was used as a neobladder (gastrocystoplasty) in experimental dogs and the remaining GI tract was rerouted via gastroduodenostomy, a high rate of morbidity and mortality was reported [58]. In a later study on experimental dogs, an isolated flap of the body of the stomach was used as a bladder substitute (with a small residual amount of native bladder trigone remaining), which resulted in reduced urine pH after meals and erosions/ulcerations in the bladder remnant in all six dogs [57]. Denuding the GI mucosa has subsequently been performed in an effort to reduce mucosal secretion/absorption and the subsequent metabolic changes, as well as to provide a submucosal surface over which transitional bladder epithelium can develop [59].

Segments of the small intestine have also been used as a bladder substitute. One study used jejunum to create a jejunocystoplasy following cystectomy and subtotal intracapsular prostatectomy in eight healthy male intact dogs [60]. A segment of jejunum was mobilized with preservation of its blood supply, closed at one end, and anastomosed to the remaining urethra/prostatic remnant at the other end, with bilateral ureteral implantation also performed [60]. Complication rates were high and included complete ureteral obstruction requiring a second surgery, pyelonephritis, epididymitis, and substantial pollakiuria despite maintaining urinary continence; the authors concluded that the outcomes of these dogs were unacceptable for most household pets [60]. Another study described modified ‘cup-patch’ ileocystoplasty to treat bladder necrosis in a young female dog 2 days after ovariohysterectomy [61]. A full thickness incision was performed on the antimesenteric surface of an isolated, vascularized segment of ileum and all mucosa was denuded [61]. The open segment of ileum was doubled on itself to form a “U”, and the apposing edges sutured together to form a vesicle (with submucosa internally) for the neobladder [61]. The ureters were implanted into the vesicle, and the neobladder was anastomosed to the urethra [61]. The procedure was overall well tolerated; renal function testing, urinalysis, and urine cultures were considered normal on postoperative assessments [61]. The dog’s bladder capacity gradually increased over 1 year, and 6 months postoperatively, the dog had normal urination frequency and was continent [61]. A final report documented use of jejunocystoplasty in a dog following total cystectomy that was performed as a result of surgical error during caesarian section and ovariohysterectomy for treatment of dystocia [62]. A similar technique for jejunocystoplasty was reported as per the Schwarz et al. ileocystoplasty, though one ureter was implanted into the neobladder with stent placement and the other ureter was implanted into the urethra [61,62]. Postoperative complications included retention of the ureteral stent, persistent pyelectasia and hydroureter, recurrent urinary tract infections, and intermittent urinary incontinence [62].

One additional study evaluated the use of a silicone modeler in experimental dogs undergoing total cystectomy and ileocystoplasty [59]. Following total cystectomy and bladder replacement with a neobladder of demucosalized ileal segment, an empty silicone modeler was inserted and inflated with 100 mL physiologic saline solution, with a valve placed in the subcutaneous tissues of the abdominal wall; the silicone modeler was removed 14 days postoperatively [59]. Postoperatively, 2/12 dogs that underwent ileocystoplasty died within 1 month due to uroabdomen [59]. Placement of the intravesical silicone modeler prevented retraction of the neobladder and increased bladder capacity compared to ileocystoplasty without silicone modeler placement [59].

Although several reports document adequate outcomes with enterocystoplasty techniques in dogs undergoing total cystectomy, the risk of complications is relatively high, and consideration of alternative urinary diversion techniques should therefore be considered.

#### 3.3.2. Colonic Ureterostomy

Following total cystectomy in both experimental dogs and dogs with UC of the urinary bladder trigone/urethra, ureterocolonic anastomosis has been described for urinary diversion [63,64,65]. Following total cystectomy, a submuscular flap was prepared in the distal colon and an end-to-side anastomosis of the ureteral to colonic mucosa was created for each ureter using 6-0 absorbable suture in a simple interrupted pattern, with subsequent loose reattachment of the submuscular flap to form a submuscular tunnel for each ureter [63,64]. Red rubber catheters were placed through the ureterocolonic anastomoses as stents in several dogs [63]. The remaining urethral defect was closed. Postoperatively, a rectal catheter (multi-holed 8 Fr tube) was placed to drain urine in several cases, and broad-spectrum antibiotics were administered for at least 1 month [63]. In clinically normal dogs, variable degrees of metabolic acidosis, hyperammonemia, and neurologic disease occurred [64]. In 10 dogs with UC, all maintained anal continence and had no urine leakage [63]. However, 4/10 dogs were euthanized due to neurologic disease, three of which also experienced nausea/vomiting, suspected to be in association with hyperammonemia, metabolic acidosis, and uremia [63]. All dogs were azotemic with elevated BUN (due to intestinal recycling of urea), 4/10 had increased serum creatinine levels, and 5/10 had hyperchloremic metabolic acidosis postoperatively [63]. Pyelonephritis occurred postoperatively in five kidneys [63]. Postoperative survival ranged from 7 days to 5 months, and six dogs had metastatic disease at the time of death [63]. One additional case report on a dog with UC of the urinary bladder that underwent total cystectomy and ureterocolonic anastomosis reported an acceptable quality of life until tumor recurrence and euthanasia 10 months postoperatively [65]. Overall, however, this technique is generally not recommended due to the relatively high risk of severe complications, and no recent reports have been described.

#### 3.3.3. Cutaneous Ureterostomy

One described technique for urinary diversion following total cystectomy in dogs with urinary bladder neoplasia involves ureteral implantation through the body wall and skin [13,66]. For this method, following distal ureteral transection, the ureters are gently dissected from surrounding fat and mobilized from the retroperitoneal space with caution to preserve the ureteral blood supply. In one case report, the authors described cauterizing both ureteral arteries to prevent hematomas that could cause stricture of the ureterostomy sites [13]. The ureters can also be temporarily catheterized with small (~4 Fr) tubes and held with stay sutures for manipulation during ureterostomy. Short (3–4 cm) oblique tunnels oriented parallel to the linea alba are created bilaterally in the body wall lateral to the rectus abdominal muscle by using a scalpel blade or a 4 mm punch biopsy. A cutaneous exit site near the 4th nipple has been recommended to allow for coverage by diaper application and to reduce inguinal skin excoriation [13]. Each ureter is then passed through the body wall and exited in the subcutaneous tissues. The serosa of each ureter is subsequently secured to the abdominal wall musculature with two simple interrupted monofilament absorbable sutures (5-0 polydioxanone). Both ureters are spatulated for 2–5 mm and the ureteral mucosa is slightly everted and sutured to the skin with a simple interrupted pattern of monofilament absorbable suture (5-0 polydioxanone, poliglecaprone 25, or nylon), with suture bite placement at 1–2 mm from the incision edges and 2 mm apart. Abdominal closure can be performed sequentially throughout the procedure (i.e., ureteral to body wall sutures, then body wall closure, then subcutaneous and skin closure, then ureterocutaneous anastomosis) or after urinary diversion is completed. Prior to closure, the proximal urethral stump must also be ligated. In one report, each ureteral stoma was catheterized with a polyvinyl chloride catheter held in place with two simple interrupted sutures through tape fixed to the catheter, and the catheters were connected to closed urine collection systems that were maintained for 5 days postoperatively, after which time dogs were managed with absorbent diapers over the stomas [66]. Alternatively, a small amount of white petroleum ointment can be applied to the peristomal skin and the ureterocutaneous anastomoses covered by a diaper postoperatively [13]. Described long-term stoma care in both reports involved diaper changes every 8–12 h, saline/dilute chlorhexidine solution cleansing of the stoma sites, and application of white petroleum/zinc oxide-lanolin cream to the stoma sites [13,66].

A report on four dogs with trigonal UC that underwent total cystectomy and cutaneous ureterostomy documented minor complications in all dogs, including bleeding and edema of ureterostomy sites for the first 2–3 days; one dog experienced urine scald which improved with stoma care and hygiene [66]. The median survival time was 279 days postoperatively; two dogs were euthanized for reasons unrelated to surgery or UC, and two dogs had documented metastasis with subsequent euthanasia [66]. Most owners considered postoperative management and quality of life to be acceptable [66]. Another case report of a dog with extraskeletal osteosarcoma of the bladder neck underwent en bloc resection of the mass with total cystectomy, vaginectomy, and urethral resection and subsequent creation of bilateral cutaneous ureterostomies [13]. The dog had no reported postoperative complications and no evidence of recurrence or metastasis at 65 months postoperatively [13]. The owner reported slightly exacerbated atopy signs due to diaper use but was overall satisfied with quality of life despite urinary incontinence [13]. Therefore, though potential for complications exists and diligent long-term stoma management is required, this technique is a reasonable consideration for urinary diversion in dogs following total cystectomy, with adequate outcomes reported in the majority of dogs, though large-scale studies are lacking.

#### 3.3.4. Uretero-Urethral Anastomosis

Another urinary diversion technique that has been described in male dogs with UC following total cystectomy with prostatectomy involves anastomosis of the distal ureters and internalized penile urethra [54,67]. This procedure requires both abdominal and perineal approaches with the dog positioned in dorsal recumbency, and it is only applicable to male dogs due to the available length of urethra [54]. The procedure is described as follows by Bacon et al. [54]. The procedure is initiated routinely with a caudal midline/parapreputial celiotomy. Following transection of both ureters 2–3 cm proximal to the ureterovesicular junctions, stay sutures are placed and a red rubber catheter is inserted into each ureter. Total cystectomy with prostatectomy is performed with peri-urethral dissection continued as caudal as possible within the pelvis. The celiotomy incision is then extended into the perineal region and soft tissues are dissected to reveal the retractor penis muscle, bulbospongiosus, and ischiocavernosus muscles. The ischiocavernosus muscles are transected bilaterally near the ischiatic tuberosities, and dissection of the bulbospongiosus is continued into the pelvic canal, while ensuring preservation of the deep artery/vein of the penis, until the pelvic urethra is free from attachments and the prostate is visible. The penis is then dissected cranially to the level of the os penis, and following dissection to separate the dorsal penile vasculature from the penis, the penis is transected caudal to the scrotal remnant. The bladder, prostate, pelvic urethra, and portion of the penis are then removed via the celiotomy. The ureteral catheters are removed, and a 1 cm longitudinal incision is made in each distal ureter. The distal ureters are subsequently sutured together side-by-side in a simple continuous pattern with 5-0 poliglecaprone 25 to create a single wide lumen of the conjoined ureters. The transected penile remnant is diverted into the abdomen and two red rubber catheters (3 Fr) are introduced retrograde from the penis through the urethra into each ureter. An end-to-end anastomosis is performed over the catheters between the transected penile urethra and single conjoined ureteral end with simple interrupted 5-0 poliglecaprone 25 sutures. The outer penile connective tissues are anchored to the linea alba to reduce tension and maintain the anastomosis within the abdomen, and an omental patch is wrapped around the anastomosis. All catheters are then removed, and abdominal closure is routine, taking care not to compress the penis at the caudal aspect of the abdomen.

This diversion technique has been reported in three dogs with UC. All dogs had urinary incontinence postoperatively (though two dogs also were incontinent preoperatively) [54,67]. One dog developed pyelonephritis postoperatively and was euthanized at 8 months postoperatively due to progressive metastatic disease [54]. Another dog experienced multiple postoperative complications including uroabdomen with requirement for surgical revision, aspiration pneumonia, and pyelonephritis; this dog was euthanized within 2 weeks of surgery due to these complications [54]. The final dog experienced bilateral ureteral obstructions postoperatively that required surgical revision including bilateral ureteral stent placement, with subsequent complications including uroabdomen, aspiration pneumonia, and urinary tract infections [67]. However, the dog’s quality of life was considered excellent at home [67]. Local recurrence was suspected approximately 3 months postoperatively, and subcutaneous ureteral bypass (SUB) placement was performed 6 months postoperatively for treatment of obstruction [67]. The dog was euthanized 301 days after total cystectomy due to clinical signs associated with progressive urethral, ureteral, and abdominal wall UC [67]. Therefore, though limited data exists with reports in only three dogs, this urinary diversion technique can be considered following total cystectomy, but the risk of major complications appears to be relatively high and alternative urinary diversion methods should be considered.

#### 3.3.5. Ureteral Diversion to the Prepuce or Vagina/Uterus

To date, a single study has described ureteral transplantation to the prepuce following total cystectomy in male dogs with trigonal UC [53]. This technique requires complete removal of the penis and distal urethra as well as castration (if intact). Subsequently, two orifices are created in the preputial mucosa with a 2–3 mm skin punch biopsy. The location of these preputial mucosal orifices is determined by residual ureteral length and postoperative diaper coverage. The ureters are then tunneled through stab incisions in the body wall and anastomosed to the preputial mucosa with 5-0 or 6-0 absorbable monofilament suture in a simple interrupted pattern. Two dogs with UC underwent this procedure and one dog experienced a major complication characterized by uroabdomen 1 day postoperatively due to distal ureteral necrosis; revision surgery was required and ureterocolonic anastomosis was performed, with subsequent complications associated with this anastomosis [53]. This dog had evidence of metastatic disease at the time of death 433 days postoperatively [53]. The other dog experienced no significant perioperative complications, though it developed tumor recurrence and was euthanized 176 days postoperatively [53].

Several studies have described ureteral transplantation to the reproductive structures of female dogs with urinary bladder neoplasia (majority UC, one case of leiomyosarcoma) that were previously spayed or underwent ovariohysterectomy/ovariectomy at the time of diversion [12,52,53]. In these reports, the ureters were either anastomosed to one another via an end-to-side or side-to-side anastomosis with subsequent anastomosis of a single ureteral structure to the vagina, or each ureter was anastomosed to the vagina independently; ureterovaginal anastomosis was performed similarly as per ureteropreputial anastomosis [12,52,53]. Postoperative complications included oliguria attributed to acute renal failure, pyelonephritis, ureteral obstruction managed with initial nephrostomy tube placement followed by ureteral stent placement, and dehiscence of the anastomosis site which required revision surgery [53]. These dogs with UC survived 92–718 days, and 5/8 dogs had disease progression (local recurrence and/or metastasis) at the time of death/euthanasia [52,53]. One additional dog that underwent total cystectomy and ureterovaginal anastomosis for leiomyosarcoma developed postoperative necrosis of the distal ureter with subsequent dehiscence of the anastomosis; revision surgery required ureteral anastomosis to the ipsilateral uterine horn with stent placement across the uterine cervix [12]. At 12 months postoperatively, this dog developed obstruction of the stent with subsequent ureteral obstruction, and the dog died 2 months later [12].

Overall, limited data exists with reports in only two dogs following ureteral diversion to the prepuce and ten dogs following ureteral diversion to the vagina/uterus. These urinary diversion techniques can be considered following total cystectomy in male and female dogs, respectively, but the risk of major complications appears to be relatively high and alternative urinary diversion methods should also be considered.

#### 3.3.6. Urinary Diversion via Subcutaneous Ureteral Bypass (SUB)

A final described technique of urinary diversion following total cystectomy in dogs entails placement of SUB catheter devices to create communication between the renal pelves to the urethra or reproductive tract [68,69]. Following total cystectomy, SUB catheters are placed in the bilateral renal pelves, with each catheter then connected to a 3-way port, and a third catheter is placed from the port into the urethra or vagina [68]. Urinary incontinence is expected postoperatively. A single case of a female spayed dog with an extensive urinary bladder leiomyoma involving the trigone has been documented with use of this technique [69]. In this case, following total cystectomy, the ureters were concurrently excised, and the urethra was oversewn. Bilateral SUB nephrostomy catheters (6.5 Fr locking loops) were placed with fluoroscopic guidance in both renal pelves, and a 6.5 Fr non-locking loop SUB catheter was placed into the ventral aspect of the proximal urethra. These three catheters were connected via a three-way port placed in the subcutaneous tissues external to the body wall. No intraoperative complications occurred. Postoperatively, the dog was urinary incontinent with adequate urination and no evidence of azotemia. However, within 24 h postoperatively, the dog developed progressive dyspnea and oxygen dependence, with thoracic radiographs revealing suspected atypical aspiration pneumonia; antimicrobials and supportive care were administered, and the dog’s respiratory status improved. However, following an episode of regurgitation, the dog experienced cardiopulmonary arrest and was not successfully resuscitated.

Though this technique holds promise for urinary diversion following total cystectomy in dogs, long-term data is lacking such that the risk of postoperative complications and associated outcomes are not yet known.

### 3.4. Discussion

Overall, conclusions regarding total cystectomy in dogs are based on limited data available to date, predominantly characterized by small case series/reports and healthy experimental dogs. No large-scale or prospective studies are available to draw robust conclusions. However, based on the current literature, relatively high complication rates exist with many total cystectomy procedures and neobladder formation or urinary diversion techniques. However, for dogs with dedicated owners that are willing to manage these complications (potentially with revision surgeries), death/euthanasia is often attributed to progressive neoplastic disease rather than surgical complications, as surgery is rarely expected to be curative for dogs with UC. Therefore, these radical cystectomy techniques can be considered in scenarios with appropriate case and owner selection, but surgical complications and permanent urinary incontinence should be expected in addition to disease progression in the setting of canine UC.

## 4. Conclusions

In conclusion, this review provides a summary of the expansive literature encompassing cystectomy procedures in dogs. Surgical excision as a component of treatment for lower urinary tract neoplasia in dogs may enhance survival time and result in acceptable quality of life. However, for dogs with UC, cystectomy is generally not considered curative and disease progression is common. Appropriate case selection and thorough preoperative discussion with owners regarding potential risks and benefits of cystectomy are imperative for successful outcomes.

## Data Availability

Not applicable.
